# Effects of a Game-Based Virtual Reality Video Capture Training Program Plus Occupational Therapy on Manual Dexterity in Patients with Multiple Sclerosis: A Randomized Controlled Trial

**DOI:** 10.1155/2019/9780587

**Published:** 2019-04-22

**Authors:** Carmen Nélida Waliño-Paniagua, Cristina Gómez-Calero, María Isabel Jiménez-Trujillo, Leticia Aguirre-Tejedor, Alberto Bermejo-Franco, Rosa María Ortiz-Gutiérrez, Roberto Cano-de-la-Cuerda

**Affiliations:** ^1^Department of Physiotherapy, Occupational Therapy, Rehabilitation and Physical Medicine, Health Sciences Faculty, Universidad Rey Juan Carlos, Alcorcón, Madrid, Spain; ^2^Department of Medicine and Surgery, Psychology, Preventive Medicine and Public Health and Medical Microbiology and Immunology, Nursing and Stomatology, Faculty of Health Sciences, Universidad Rey Juan Carlos, Alcorcón, Madrid, Spain; ^3^Occupational Therapy Unit, Asociación Mostoleña de Esclerosis Múltiple (AMDEM), Móstoles, Madrid, Spain; ^4^Servicio de Rehabilitación, Hospital Universitario Ramón y Cajal, Madrid, Spain; ^5^Department of Physiotherapy, Centro de Estudios Universitarios San Rafael–Nebrija, Madrid, Spain

## Abstract

Neurorehabilitation is a fundamental aspect in the treatment approach for multiple sclerosis (MS), in which new technologies have gained popularity, especially the use of virtual reality (VR). The aim of this paper is to analyze an occupational therapy (OT) intervention compared with OT + VR (OT + VR) on the manual dexterity of patients with MS. 26 MS subjects were initially recruited from an MS patient association and randomized into two groups. The OT group received 20 conventional OT sessions distributed in two sessions per week. The OT + VR group received 20 sessions of VR interventions, twice weekly and lasting 30 minutes, consisting of VR games accessed via the online web page motiongamingconsole.com, in addition to the conventional OT sessions. Pre- and postintervention assessments were based on the Purdue Pegboard Test, the Jebsen-Taylor Hand Function Test, and the Grooved Pegboard Test. Clinical improvements were found regarding the precision of movements, the execution times, and the efficiency of certain functional tasks in the Purdue Pegboard Test and Jebsen-Taylor Hand Function Test tests in the OT + VR group. Although significant differences were not found in the manual dexterity between the OT and OT + VR groups, improvements were found regarding the precision and effectiveness of certain functional tasks.

## 1. Introduction

Multiple sclerosis (MS) is a chronic inflammatory demyelinating illness of the central nervous system (CNS) of unknown etiology, currently representing the most common neurological illness causing disability among young adults in Europe and North America [1]. Common symptoms include fatigue, visual disorders, problems affecting balance and coordination, sensitivity disorders, spasticity, cognitive and emotional disorders, speech disorders, problems affecting the bladder and intestines, and sexual-related dysfunction [2].

Different disease courses exist for MS, according to the appearance of symptoms, characterized by relapses or flare-ups, which vary from one episode to the other, according to the affected CNS region. The different types of MS include relapsing-remitting MS, primary progressive MS, secondary-progressive MS, and progressive-recurrent MS [3]. Relapsing-remitting MS is the most common form of MS, whereas progressive-recurrent MS is the least common type of illness.

The treatment of MS commonly features both pharmacological and rehabilitation treatments. Rehabilitation programs can increase the effectiveness of pharmacological treatment by providing symptomatic treatment of MS to improve the quality of life and functional independence of affected individuals. The main therapeutic demands are the alterations of postural control and the performance of activities of daily living (ADLs) [4–6]. Occupational therapy (OT) evaluates the capacities and physical, psychological, sensory and social problems of individuals with MS, to support their independence in daily living and/or to facilitate adaptation to their disability [7].

At times, rehabilitation treatments for patients with MS can be very lengthy and systematic, leading to loss of motivation and compliance. As a result, in recent years, new intervention strategies have been introduced, such as virtual reality (VR), thanks to VR motion capture technology, without requiring a device or controller. These novel approaches enhance patient motivation by enabling the practice of functional tasks in virtual surroundings, providing patient feedback concerning results, all of which is based on the repetition of ADLs. Thus, rehabilitation professionals have expanded the care of patients with MS, by including this technology as a complement to rehabilitation programs, achieving a higher treatment intensity at a sustainable cost [8]. However, few studies exist on the effects that VR has on the manual dexterity of patients with MS [9–11]. Thus, the aim of this study was to analyze the effects of an OT intervention combined with VR on manual skills, compared with conventional OT approaches in people with MS.

## 2. Methods

### 2.1. Study Design

We conducted a single-blinded randomized controlled trial (RCT). Nonprobabilistic sampling of consecutive cases was used. The sample was divided into a control group (OT) who received conventional OT treatment and an experimental group (OT + VR) who received VR treatment in addition to their conventional treatment sessions. All interventions were performed at the Mostoleña Association of Multiple Sclerosis (AMDEM) in Madrid (Spain).

The study inclusion criteria were as follows: a diagnosis of MS according to the McDonald criteria [2] with over two years evolution; a score of between 3.5 (moderate incapacity, although totally ambulant, self-sufficient, and active during 12 hours/day) and 6 (requires constant help, either unilaterally or intermittently with a walking stick or crutches, in order to walk approximately 100 meters with, or without, a rest) on the Kurtzke Expanded Disability Status Scale (EDSS); with stable medical treatment during at least the six months prior to the intervention; muscle tone in the upper limbs not greater than two points on the modified Ashworth Scale (moderate hypertonia, increased muscle tone through most of the range of movement, but affected part easily moved); as well as a score of four points or less in the “Pyramidal Function” section of the EDSS functional scale; absence of cognitive decline; with the ability to understand instructions and obtaining a score of 24 or more in the Mini-Mental Test; and a score of two points or less in the “Mental Functions” section of the EDSS.

The exclusion criteria were a diagnosis of another neurological illness or musculoskeletal disorder different to MS; the diagnosis of a cardiovascular, respiratory, or metabolic illness or other conditions which may interfere with the study; suffering a flare-up or hospitalization in the last three months prior to commencement of the assessment protocol or during the process of the therapeutic intervention; receiving a cycle of steroids, either intravenously or oral, six months prior to the commencement of the assessment protocol and within the study period of intervention; receiving treatment with botulinum toxin in the six months prior to the beginning of the study; or the presence of visual disorders noncorrected by optical devices.

All participating subjects voluntarily signed an informed consent form. The present study was approved by the Research Ethics Committee of the Rey Juan Carlos University (Ref 26/12).

### 2.2. Participants

**Figure 1:**
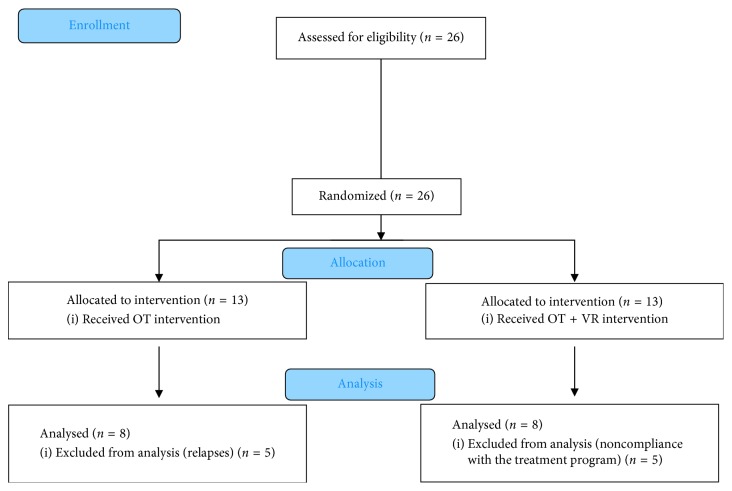
Flowchart diagram.

Twenty-six subjects with relapsing-remitting MS were initially recruited and randomized into two groups by tossing a coin. Thereafter, 10 subjects could not complete the study due to relapses or noncompliance with the treatment program. Finally, the control group (OT) comprised eight participants (*n*=8), and the experimental group (OT + VR) also comprised eight participants (*n*=8) (Figure 1).

### 2.3. Intervention

Conventional OT treatment consisted of 20 sessions during which subjects performed activities for training manipulative and functional dexterity of the upper limb aimed at ADLs. These were distributed in two OT sessions per week, each lasting 30 minutes.

The intervention applied to the experimental group consisted of 20 sessions of conventional OT distributed in two sessions per week, each lasting 30 minutes. Additionally, they received 20 treatment sessions lasting 20 minutes, twice weekly of VR via the online and free website motiongamingconsole.com, during which they performed exercises with video capture of the upper limb movements via the performance of functional and manual dexterity activities based on the following games: Flip Out, Air Hockey, Particles, DunkIt, Counting Fish, and Robo Maro. There were not used a hand controller or armbands. All exercises were designed to promote specific practice of movements in the shoulder, elbow, wrist, and/or hand through games displayed on a computer. OT + VR sessions included leisure activities such as playing cards, hitting a hockey puck, moving particles through a virtual scenario avoiding colliding with other elements, fishing, and playing “Jenga”. Patients were instructed to remain in a sitting position and use both upper limbs in these activities. All tasks present a timer as a visual feedback.

All OT and OT + VR interventions were performed by two occupational therapists, one for each modality, experts on MS neurorehabilitation. All interventions considered the level of fatigue experimented by each patient based on a progressive increase in treatment times according to the same.

### 2.4. Outcome Measures

All assessments were performed by physical therapists trained in the use of the measures and blinded to the intervention received by the subjects. The following outcome measures were used in both groups, both at the beginning and at the end of the intervention.

The *Purdue Pegboard Test* (PPT) [12, 13] was used for the assessment of fine manual dexterity, gross dexterity, and coordination. This test evaluates the speed and motor dexterity of each hand and the manual dexterity using both hands at the same time. The PPT features a board with two columns with 25 holes each and a specific number of pins, washers, and collars placed in four containers across the top of the board. The test consists of inserting as many pins as possible in three distinct phases, with a time limit of 30 seconds for each part. First, the test is performed with the dominant hand, then with the nondominant hand, and then with both hands at the same time. The number of pins inserted is recorded.

The *Jebsen-Taylor Hand Function Test* (JTT) [14] was used to determine the hand's functional capacity. This test is timed and divided in seven parts. The seven subtests are writing, page turning, picking up small common objects, simulated feeding, stacking checkers, moving large light objects, and moving large heavy objects.

All the subtests are performed with the nondominant hand first, followed by the dominant hand. The time the subject takes to perform each subtest is recorded.

The *Grooved Pegboard Test* (GPT) [15] is a test that evaluates manipulative dexterity. This test is performed with the dominant hand and consists of inserting pegs in the slots of a board which are placed at different angles. The score is the time in seconds required for inserting all the pegs [16].

All the data were introduced into the SPSS v.17.0 statistical package. A descriptive analysis of the quantitative variables was performed using measures of central tendency and dispersion measures: mean ± standard deviation (SD) and range. The pre-post comparison of each group and the comparisons between the control and experimental group were performed via the nonparametric Wilcoxon and Mann–Whitney *U* tests, respectively, as the data did not follow a normal distribution. The level of statistical significance was set at *p* < 0.05.

## 3. Results

16 patients (8 males and 8 females) successfully completed the study. The mean age of subjects was 46.44 years (SD 9.09). Concretely, in the control group (4 males and 4 females), the mean age was 46.13 years (SD 9.49), and in the experimental group (4 males and 4 females), it was 46.75 years (SD 9.31). The age range in the OT group was 32–61 years, and in the OT + VR group, it was 33–62 years. For the totality of the sample, the dominant hand was the right in 62.5% of subjects. Regarding change in dominance (patients who had to change their dominance to the other hand due to impairment), in 25%, the dominant hand prior to the appearance of MS was the left, and for 75% of the sample, it was the right.

Participants from both study groups attended 100% of the proposed sessions in both protocols. No adverse effects were registered.

### 3.1. Intragroup Pre-Post Comparison

Regarding the pre-post intervention data for the PPT, in the case of the control group (Table 1), a greater number of total pins were registered in the postintervention assessment, although statistically significant data were not obtained (*p* > 0.05). Regarding the differences in the JTT in the control group (Table 2), statistically significant differences were found regarding “Writing with the nondominant hand” (*p*=0.018) and “Picking up small common objects with the dominant hand” (*p*=0.012). Besides, improvements were observed regarding the execution time of tasks, although these values did not reach the level of statistical significance (Table 2). On the contrary, in the GPT, the control group increased the final mean scores in the number of correctly placed pieces using the dominant and nondominant hand, as well as the execution time and the number of pieces picked up and placed with the dominant hand, although these values did not reach statistical significance (Table 3).

Table 1 shows the PPT pre-post intervention scores for the experimental group. A slight decrease in the number of inserted pins was observed; however, the results do not appear statistically significant. Table 2 displays the pre-post intervention data for the JTT test obtained by the experimental group. Statistically significant changes were found in the tasks “Picking up small common objects” with the nondominant hand (*p*=0.036) and the dominant hand (*p*=0.017). A tendency towards statistical significance was observed for the task “Page turning” with the dominant hand. Table 3 features the pre-post intervention data for the GPT test in the experimental group. Statistically significant differences were found in the item “number of correctly placed pegs” with the nondominant hand (*p*=0.078). Furthermore, improvements were found in the times of the nondominant hand at the end of the intervention; however, these results were not significant (*p* > 0.05). Also, there was an increase in the correct placement of pegs and in the number of pegs fallen and placed with both hands, without this being statistically significant.

### 3.2. Intergroup Pre-Post Intervention Comparisons

The intergroup comparisons for the PPT revealed no statistically significant differences (*p* > 0.05) (Table 1). Table 2 displays the intergroup comparisons for the JTT. No statistically significant differences were found for any of the variables (*p* > 0.05) (Table 2). The intergroup comparison of the GPT also failed to reveal statistically significant results (*p* > 0.05) (Table 3).

## 4. Discussion

Our findings reveal that significant differences do not exist in the treatment of manual dexterity in subjects performing the OT + VR intervention when compared to those receiving conventional OT treatment. However, statistically significant differences were found in items such as “Picking up small common objects” using the nondominant hand and the dominant hand, with a tendency towards statistical significance in the case of “Number of correctly placed pegs” in the OT + VR group. Furthermore, several variables related to effectiveness and motor dexterity also showed a tendency towards statistical significance in both groups.

Regarding the conventional OT intervention, statistically significant differences were observed in the JTT test for the following items: “Writing” in the nondominant hand and “Picking up small objects” with the dominant hand. To our knowledge, this study is the first to evaluate manual dexterity in a population of MS, using the JTT. The results obtained may be due to the therapeutic approach of OT in patients with MS, based on the performance of functional activities with the upper limb, as well as training the change in hand dominance to enable a greater participation in ADLs [3].

Concerning the combined OT + VR interventions, the number of pins inserted between the initial and final assessments was maintained in the PPT test. Gallus et al. [17] identified the PPT as a valid measure for evaluating fine motricity and gross coordination in people with MS. In the JTT test, significant changes were observed in the tests “Picking up small common objects” with the dominant and nondominant hand, as well as a tendency towards statistical significance in the “Page turning” item. In the GPT test, improvements that were close to statistical significance were found in the number of correctly placed items. Possibly, the limitation of the sample size may have influenced these results. In the scientific literature, we were unable to find studies related with the assessment of motor dexterity via the application of the JTT and the GPT in people with MS. However, Lozano et al. [11] used the JTT in people with brain damage, finding a clinical and significant improvement in the performance of daily functional tasks such as “Page turning” and “Picking up small common objects”, using low-cost virtual reality surroundings with video capture of movement using the Kinect system. The results of the cited study coincide with our findings based on a free online games platform used in which the upper limb movements of patients with MS were registered during the performance of functional tasks. Given the context of our study, taking place at a patient association, the online platform may be interesting for situations in which there may be insufficient economic resources to enable the acquisition of new equipment. On the contrary, Merians et al. [18] also used the JTT to evaluate the fine motor dexterity of patients with brain damage as a measure of results after the VR intervention, finding clinical improvements in the speed and precision of fine movements, and in some subjects, a post-intervention generalization of learning to ADLs. These data are in line with our findings, in which clinical improvements existed, without achieving statistical significance, possibly due to the reduced sample size after the losses experienced during the study. It is well-known that the performance of functional tasks, repeated over time and with certain variability, can lead to a relearning of skills. This is an aspect reinforced by VR by offering feedback of results in real time [17, 19, 20].

We found no differences between the application of OT and OT + VR on the manual dexterity of MS patients with a moderate level of impairment in the PPT, JTT, and GPT tests. However, clinical improvements were found after the OT + VR intervention, with improved precision of the upper limb movements, faster performance. and a greater efficiency in the performance of certain functional tasks. Previous qualitative studies [21] on the subjective experience of using VR systems, based on video capture of movement with Kinect as a therapeutic tool in patients with MS, have identified improvements in patient's self-efficacy for management of the illness, social support, expectations, and training offered, as well improvements in the behavior and perception of the person's own identity, and a positive association between the physical activities performed with VR and the real environment. These results have been confirmed in similar studies [22, 23] highlighting the potential usefulness of these low-cost systems as a complement to conventional approaches from the perspective of MS patients.

As previously mentioned, we were unable to find previous scientific studies associating improvements in manipulative dexterity using OT treatment approaches combined with VR in patients with MS. Shin et al. [24] found clinical improvements in manipulative dexterity after a VR intervention in people with brain injury, measured using the PPT and JTT. Significant differences were however not found in the cited study among the group receiving conventional OT, leading the authors to conclude that the combination of conventional OT with VR may improve global upper limb movements. Our results contrast partially with those by Shin et al. as an improvement seems to exist in our OT group, as well as the OT + VR group, although significant differences were not found between both study groups. This suggests that both approaches could be valid, and, fundamentally, complementary. Findings by our research group [17, 19, 20], as well as those reported by other authors [25], have shown improvements in postural control, optimization of the processing of the sensory information, and integration of the systems necessary for maintaining balance and postural control in people with MS via the use of low-cost VR systems. Therefore, future research lines could employ whole body exercise programs with subjects in different lying positions to enhance a potential generalization of learnings to other contexts and situations in which the patient may require manual dexterity.

This study has several methodological limitations. We used a small sample size, which hampers the detection of statistically significant differences, although these may well exist. Furthermore, a high number of losses occurred due to the fluctuating nature of the illness. Also, the outcome measures used had not been previously employed in the clinical context of MS; therefore, despite their good psychometric properties, this has hampered the discussion of results regarding the manipulative dexterity in patients with MS. Lastly, future studies should consider assessments with midterm follow-up.

## 5. Conclusions

Our results show that there are no significant differences regarding manual dexterity when comparing a conventional OT intervention with an OT + VR intervention in patients with MS with a moderate level of severity. However, patients receiving an OT + VR intervention showed clinical improvements in the precision of certain upper limb movements, faster execution times for certain tests, and greater effectiveness during certain functional tasks. Therefore, VR using video capture of upper limb movements could be a complementary intervention to OT in the treatment of manual dexterity in patients with MS.

## Figures and Tables

**Table 1:** Differences pre-post intervention in Purdue Pegboard Test (PPT) in the control group and experimental group.

PPT	OT			OT + VR			*p*
	Pretreatment	Posttreatment	*p*	Pretreatment	Posttreatment	*p*	
	Mean ± SD	Mean ± SD		Mean ± SD	Mean ± SD		
DH	6.25 ± 3.65	7.50 ± 4.07	0.319	7.50 ± 4.07	7.37 ± 3.37	0.792	0.832
NDH	5.25 ± 3.57	4.00 ± 2.56	0.263	4.00 ± 2.56	4.25 ± 2.25	0.48	0.707
Bilateral	3.54 ± 2.11	3.62 ± 1.99	1	3.62 ± 1.99	3.37 ± 2.06	0.577	0.665
Assemble	2.57 ± 1.27	3.00 ± 1.63	0.518	3.00 ± 1.63	2.50 ± 2.22	0.785	0.448
Total number of pins	17.61	18.12	0.898	18.12	17.49	0.602	

DH: dominant hand; NDH: nondominant hand. Time in seconds.

**Table 2:** Differences pre-post intervention in Jebsen-Taylor Hand Function Test (JTT) in the control group and experimental group.

JTT	OT			OT + VR			*p*
	Pretreatment	Posttreatment	*p*	Pretreatment	Posttreatment	*p*	
	Mean ± SD	Mean ± SD		Mean ± SD	Mean ± SD		
Writing NDH^*∗*^ (time)	93.25 ± 73.68	62.92 ± 42.92	**0.018**	62.92 ± 42.92	50.68 ± 39.57	0.866	0.655
Page turning NDH (time)	7.98 ± 3.75	6.34 ± 2.30	0.889	7.80 ± 4.01	8.87 ± 3.25	0.208	0.248
Picking up small common objects NDH (time)	13.22 ± 6.09	10.62 ± 4.15	0.779	16.24 ± 10.08	16.73 ± 10.19	0.327	0.6
Simulated feeding NDH (time)	32.70 ± 32.66	17.75 ± 8.50	0.779	26.79 ± 19.09	26.34 ± 19.87	0.779	0.793
Stacking checkers NDH (time)	10.68 ± 10.88	6.76 ± 5.78	0.674	13.90 ± 17.87	22.91 ± 35.00	0.208	0.294
Moving large light objects NDH^‡^ (time)	8.15 ± 5.03	5.78 ± 2.05	0.08	6.98 ± 3.38	8.89 ± 5.71	**0.036**	0.345
Moving large heavy objects NDH (time)	6.84 ± 2.67	5.39 ± 1.13	0.779	7.06 ± 2.20	7.64 ± 2.32	0.327	0.4
Writing DH (time)	39.99 ± 21.68	38.40 ± 24.66	0.674	38.40 ± 24.66	37.21 ± 23.44	0.674	0.834
Page turning DH (time)	8.00 ± 2.75	6.34 ± 2.30	0.093	6.34 ± 2.30	8.14 ± 3.16	0.069	0.208
Picking up small common objects DH (time)	12.26 ± 2.14	10.62 ± 4.15	0.208	10.62 ± 4.15	13.02 ± 5.25	0.263	0.529
Simulated feeding DH (time)	16.39 ± 4.84	17.75 ± 8.50	0.484	17.75 ± 8.50	19.09 ± 7.33	1	0.529
Stacking checkers DH (time)	7.84 ± 4.47	6.76 ± 5.78	0.401	6.76 ± 5.78	8.15 ± 3.76	0.123	0.208
Moving large light objects DH^*∗*‡^ (time)	6.12 ± 1.66	5.78 ± 2.05	**0.012**	5.78 ± 2.05	6.45 ± 1.87	**0.017**	0.294
Moving large heavy objects DH (time)	6.11 ± 1.30	5.39 ± 1.13	0.208	5.39 ± 1.13	7.01 ± 1.93	0.263	0.093

^*∗*^The difference between pretreatment and posttreatment in the control group is statistically significant. ^‡^The difference between pretreatment and posttreatment in the experimental group is statistically significant. DH: dominant hand; NDH: nondominant hand. Time in seconds.

**Table 3:** Differences pre-post intervention in Grooved Pegboard Test (GPT) in the control group and experimental group.

GPT	OT			OT + VR			*p*
	Pretreatment	Posttreatment	*p*	Pretreatment	Posttreatment	*p*	
	Mean ± SD	Mean ± SD		Mean ± SD	Mean ± SD		
Time NDH	339.12 ± 277.94	340.25 ± 276.80	0.686	340.25 ± 276.80	336.18 ± 277.73	0.715	0.955
Number of fallen pegs (and collected to replace) NDH	3.71 ± 2.98	4.85 ± 3.62	0.245	4.85 ± 3.62	5.37 ± 4.56	0.336	0.861
Number of correctly placed pegs NDH	20.14 ± 8.47	21.14 ± 4.63	0.465	15.37 ± 9.67	21.14 ± 4.63	0.078	0.239
Time DH	203.52 ± 83.98	185.40 ± 58.03	0.499	185.40 ± 58.03	205.58 ± 74.64	0.237	0.674
Number of fallen pegs (and collected to replace) NDH	3.37 ± 3.20	2.50 ± 1.92	0.246	2.50 ± 1.92	3.25 ± 3.41	0.226	0.915
Number of correctly placed pegs NDH	23.75 ± 3.53	24.00 ± 2.82	0.317	24.00 ± 2.82	22.75 ± 4.30	0.18	0.538

DH: dominant hand; NDH: nondominant hand. Time in seconds.

## Data Availability

All data used to support the findings of this study are included within the article.
